# When Stones Strike Twice: A Rare Case of Post-endoscopic Retrograde Cholangiopancreatography (ERCP) Gallstone Ileus

**DOI:** 10.7759/cureus.91365

**Published:** 2025-08-31

**Authors:** Montasir Elmobark, Mohamed Mohamed, Mohamed Elzober, John Hancock, Vikramjit Mitra

**Affiliations:** 1 Gastroenterology, University Hospital of North Tees, Stockton-on-Tees, GBR

**Keywords:** choledocholithiasis management, enterolithotomy, ercp complications, gall stone, gallstone ileus post-cholecystectomy

## Abstract

Gallstone ileus is a rare complication, typically resulting from a cholecystoenteric fistula. Its occurrence following endoscopic retrograde cholangiopancreatography (ERCP), particularly without evidence of fistula formation, is exceedingly uncommon. We present the case of a 69‑year‑old man with choledocholithiasis who developed gallstone ileus 48 hours after successful ERCP and stone extraction. The risk of gallstone ileus was anticipated during the procedure due to the large size of the bile duct stones. The patient subsequently developed symptoms of small bowel obstruction and underwent surgical intervention with enterolithotomy. Recognition of this potential complication, even in the absence of a fistula, is critical for timely diagnosis and management. This case emphasizes the need for careful procedural planning, documentation, and post‑procedural vigilance in patients with large biliary stones.

## Introduction

Endoscopic retrograde cholangiopancreatography (ERCP) is a well-established diagnostic and therapeutic intervention for biliary and pancreatic disorders. ERCP involves endoscopic cannulation of the biliary tree via the duodenum, injection of contrast to delineate ductal anatomy, and therapeutic maneuvers such as sphincterotomy and stone extraction, each of which carries distinct risks. Although generally safe, ERCP carries potential complications, including pancreatitis, hemorrhage, perforation, and cholangitis, with complication rates reported between 4% and 10% depending on patient and procedural risk factors [1].

Gallstone ileus is an uncommon and often overlooked cause of mechanical small bowel obstruction. It accounts for less than 0.5% of all intestinal obstructions and predominantly affects elderly patients, especially females [2]. Traditionally, it results from a cholecystoenteric fistula that allows a large gallstone to enter the gastrointestinal tract, ultimately becoming lodged in areas of anatomical narrowing, typically the terminal ileum [3,4].

However, in rare instances, gallstone ileus may occur following ERCP when large stones are inadvertently advanced into the duodenum and small intestine during stone extraction, even in the absence of a fistula [5]. This unusual mechanism is rarely documented, and awareness remains limited. Here, we present a rare case of post-ERCP gallstone ileus that developed despite complete ductal clearance and no evidence of a fistula. Importantly, the risk was anticipated at the time of ERCP, reinforcing the importance of procedural foresight and vigilant follow-up.

## Case presentation

A 69-year-old man with a history of hypertension and Graves’ disease presented with right upper quadrant pain and deranged liver function tests. Laboratory investigations at presentation are summarized in Table 1.

**Table 1:** Laboratory investigations at first presentation ALT: alanine transaminase; ALP: alkaline phosphatase; GGT: gamma-glutamyl transferase/transpeptidase

Test	Result	Unit	Reference range*
Total bilirubin	82	µmol/L	0-21 µmol/L
ALT	82	U/L	≤40 U/L
ALP	338	U/L	≤120 U/L
GGT	1028	U/L	10-70 U/L

**Figure 1:**
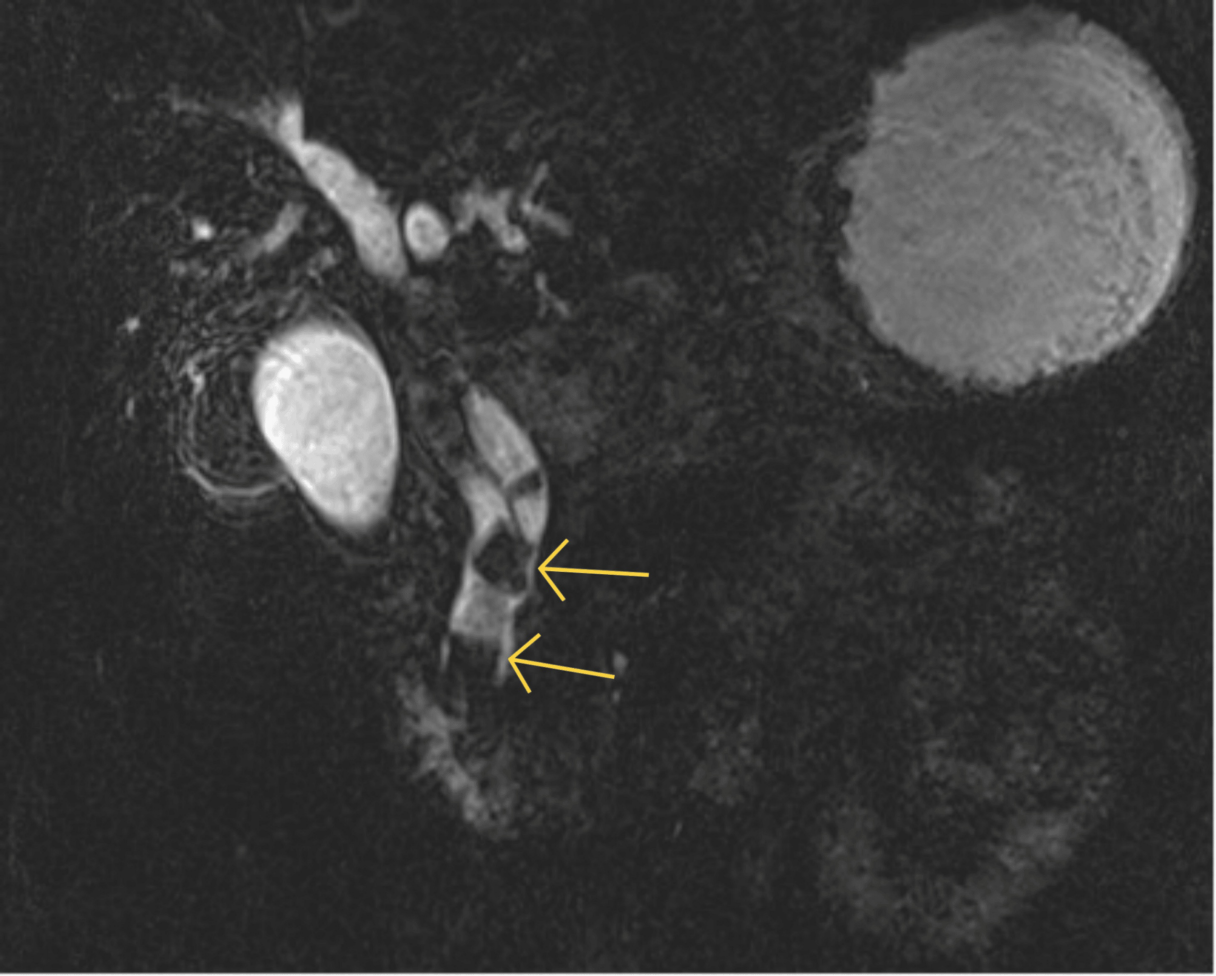
MRCP MRCP: magnetic resonance cholangiopancreatography Imaging demonstrates a markedly dilated common bile duct (CBD) measuring 20 mm, containing multiple large calculi up to 23 mm. A distal cystic duct calculus is also present, with a low and medial insertion (yellow arrows)

Abdominal ultrasound showed gallstones and a dilated common bile duct (CBD). Magnetic resonance cholangiopancreatography (MRCP) confirmed choledocholithiasis with multiple large stones (up to 20 mm) and no evidence of cholecystoenteric fistula (Figure 1).

**Figure 2:**
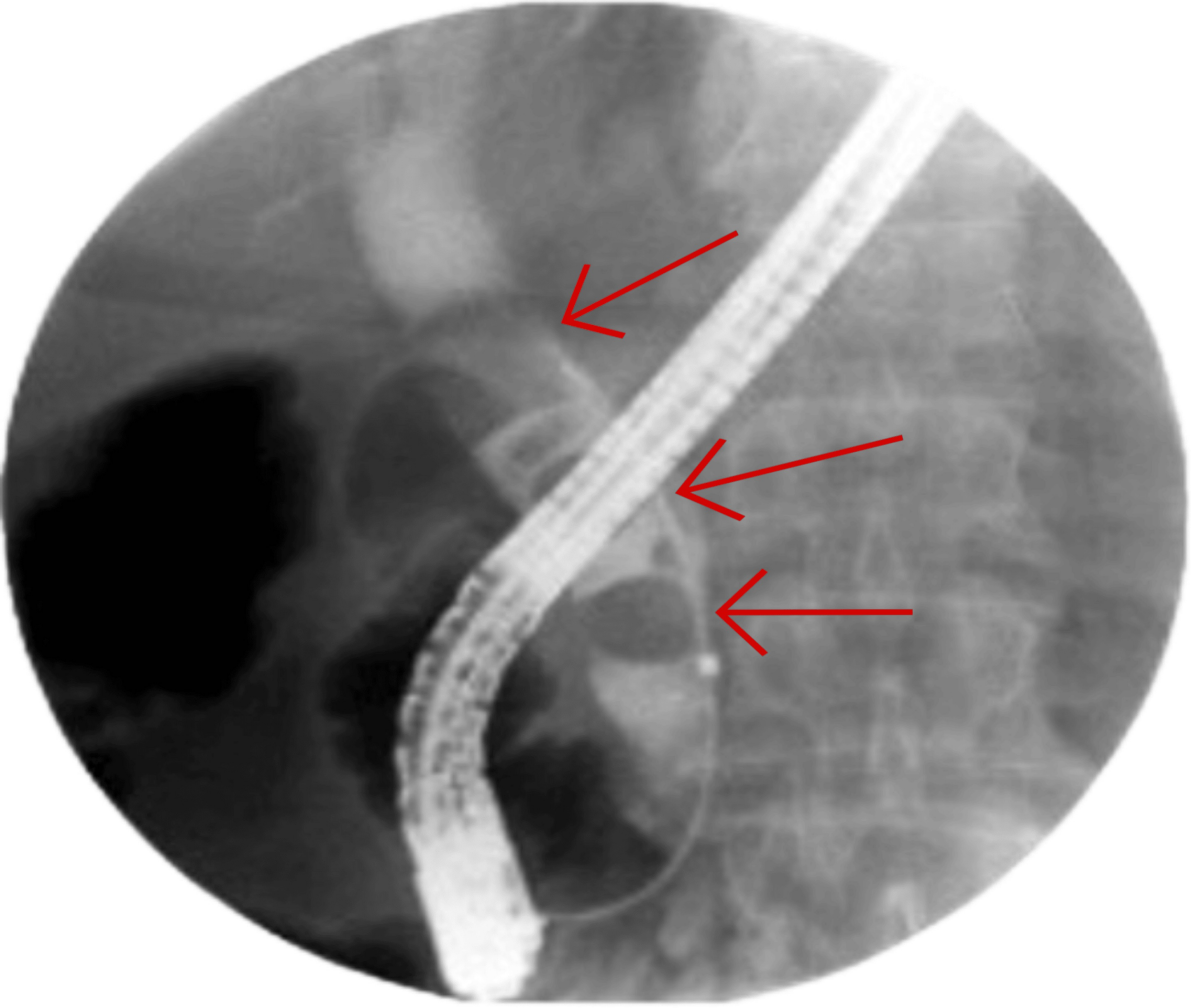
Cholangiogram Cholangiogram showing multiple stones (red arrows)

The patient underwent ERCP. Using a 0.035-inch guidewire and Fusion® OMNI™ sphincterotome, selective biliary cannulation was achieved. Cholangiogram demonstrated a dilated CBD with multiple large filling defects (Figure 2).

**Figure 3:**
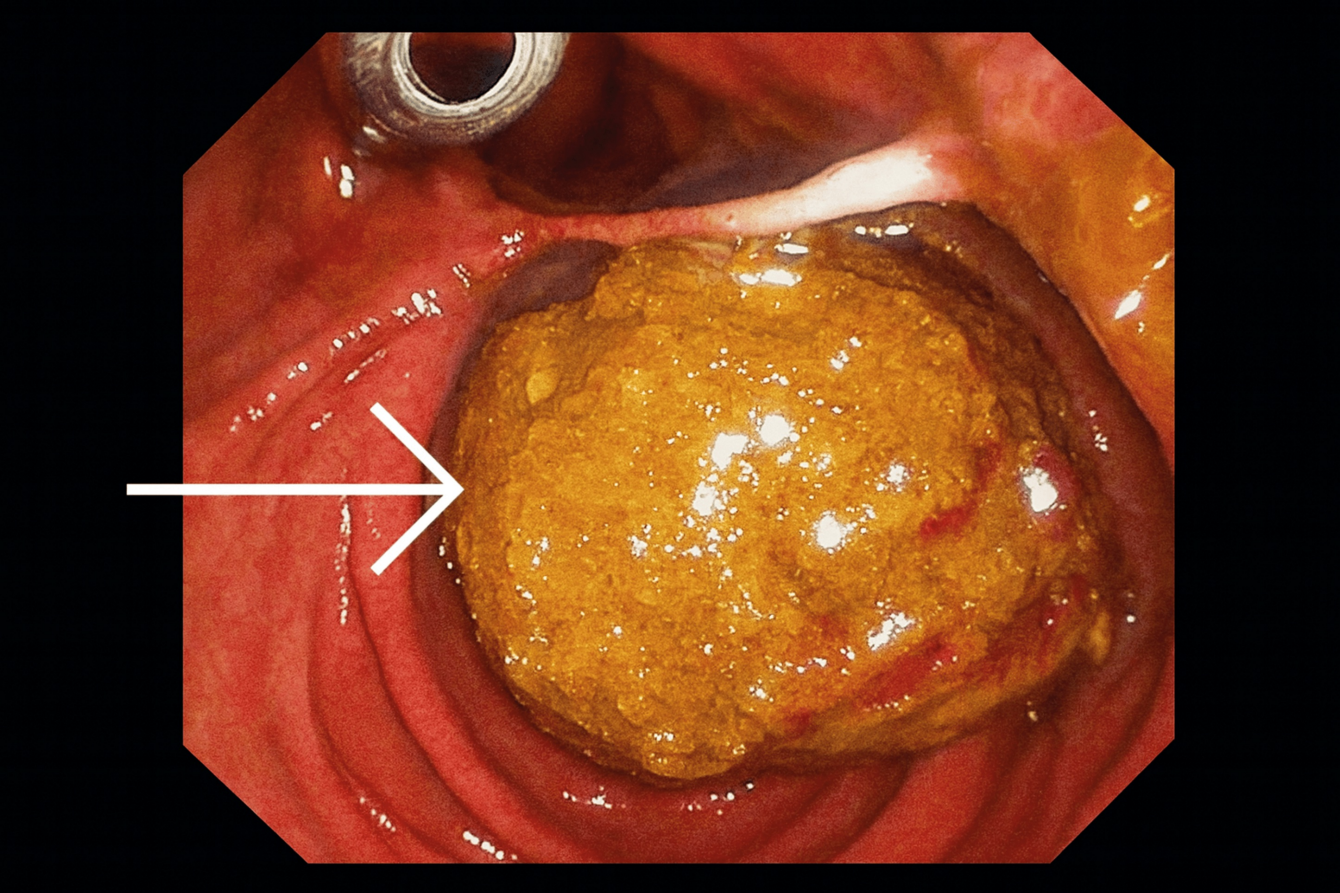
ERCP stone extraction ERCP: endoscopic retrograde cholangiopancreatography Imaging demonstrating a removal of a large stone (white arrow)

Sphincterotomy was performed, followed by multiple balloon trawls. Six large stones (the largest approximately 18 mm) were extracted successfully (Figure 3). Final cholangiogram confirmed complete ductal clearance. Due to the size and number of stones, the endoscopist documented a risk for post-procedural gallstone ileus, despite no fistula being evident.

The patient was discharged in stable condition. However, 48 hours later, he returned with colicky abdominal pain, nausea, vomiting, and abdominal distension. He remained hemodynamically stable. On examination, bowel sounds were sluggish, and the abdomen was tender but without peritonism. Repeat laboratory investigations are also shown in Table 2.

**Table 2:** Laboratory investigations at second presentation ALT: alanine transaminase; ALP: alkaline phosphatase; GGT: gamma-glutamyl transferase/transpeptidase

Test	Result	Unit	Reference range*
Total bilirubin	28	µmol/L	0-21 µmol/L
ALP	134	U/L	≤120 U/L
GGT	380	U/L	10-70 U/L
CRP	202	mg/L	<5 mg/L
Amylase	Normal	U/L	30-110 U/L

**Figure 4:**
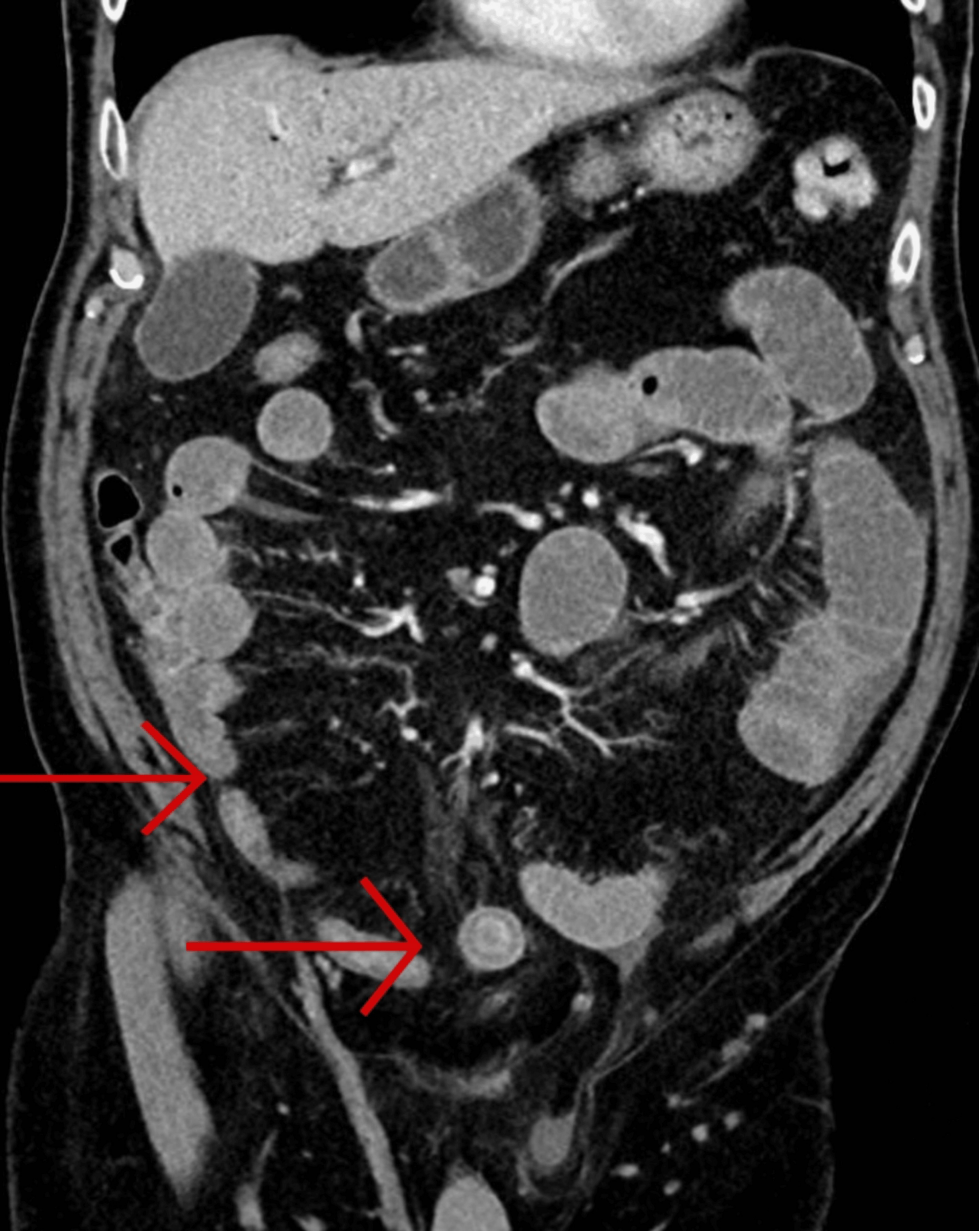
CT abdomen and pelvis with contrast Imaging demonstrating small bowel dilatation with collapsed ascending colon and large bowel with suspicion of gallstone ileus (red arrows)

CT abdomen with contrast showed dilated small bowel loops with a clear transition point approximately 30 cm proximal to the ileocecal valve, but no pneumobilia or visualized obstructing gallstone (Figure 4). However, given the recent ERCP and documented concern, gallstone ileus was strongly suspected.

**Figure 5:**
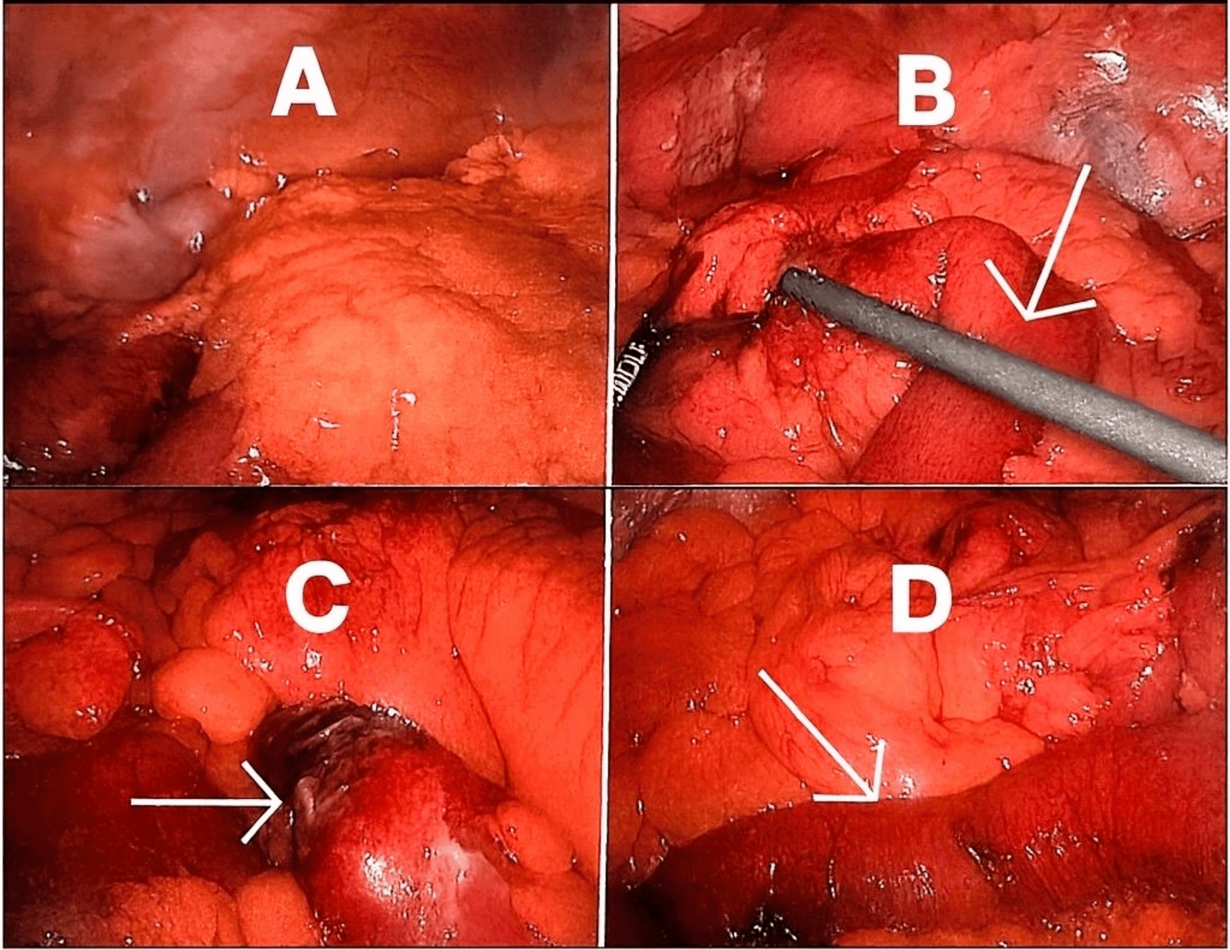
Surgical pictures during laparoscopy Surgical pictures demonstrating dusky bowel segments at the site of obstruction (white arrows at B, C, and D)

The patient proceeded to laparoscopic exploration, which was converted to open surgery. Intraoperatively, a single obstructing gallstone was found in the ileum. An enterotomy was performed, and the stone was extracted successfully. No fistula or other intra-abdominal abnormalities were identified (Figure 5). Bowel resection was not required.

The patient had an uneventful postoperative recovery and was discharged on day four. An elective cholecystectomy was scheduled.

## Discussion

Gallstone ileus is a rare form of mechanical bowel obstruction that most commonly affects elderly patients and typically results from the formation of a cholecystoenteric fistula through which large gallstones enter the gastrointestinal tract [2]. The stones usually become lodged at narrow anatomical sites, most frequently the terminal ileum [3]. Gallstone ileus occurs in approximately 0.3-0.5% of patients with gallstone disease and accounts for less than 0.5% of all intestinal obstructions [2]. Although traditionally associated with chronic inflammation and fistula formation, gallstone ileus can occasionally occur after ERCP due to the migration of large stones into the bowel lumen, even in the absence of a fistula [4]. While there are no universally accepted size thresholds beyond which endoscopic stone extraction becomes unsafe, studies suggest that stones larger than 15 mm are more likely to require advanced techniques such as mechanical lithotripsy [5]. Gallstone ileus has also been more frequently associated with stones larger than 2-2.5 cm, as these are more prone to impaction at physiologic narrowings such as the ileocecal valve [2,3]. Our patient had multiple large stones, the largest measuring 23 mm, placing him at a higher risk for post-procedural gallstone ileus.

ERCP is an established treatment for choledocholithiasis, with complication rates ranging from 4% to 10%, depending on patient risk factors and procedural complexity [1]. While common complications include post-ERCP pancreatitis, bleeding, and infection, gallstone ileus is rarely reported in this context. In our case, gallstone ileus developed within 48 hours of a technically successful ERCP with complete ductal clearance, raising the suspicion that one of the large stones had migrated distally during the procedure and later became impacted [6]. Similar rare cases of gallstone ileus following ERCP have been described in the literature [5], though the majority remain isolated case reports, underscoring the unusual nature of this complication.

Imaging studies, particularly CT, play a central role in diagnosing gallstone ileus, typically revealing pneumobilia, bowel obstruction, and an ectopic gallstone, which form the classic Rigler’s triad [6]. However, in post-ERCP cases, pneumobilia may be an expected finding, which can complicate interpretation. In our patient, the diagnosis was supported by the clinical presentation and a high index of suspicion prompted by documentation from the original ERCP. CT imaging confirmed small bowel obstruction but did not visualize the stone directly, which is not uncommon in non-calcified gallstones [6]. The classic diagnostic triad of gallstone ileus on imaging consists of pneumobilia, small bowel obstruction, and an ectopic gallstone, though in our patient only obstruction was demonstrable.

Surgical management remains the standard of care for gallstone ileus. Enterolithotomy alone is often sufficient, especially in elderly or comorbid patients. Intraoperative identification of the obstructing stone is critical, and resection is typically avoided unless ischemia or perforation is present [3]. While one-stage procedures involving enterolithotomy, cholecystectomy, and fistula repair can be performed, they are generally associated with higher morbidity, particularly in elderly patients. Enterolithotomy alone is often favored due to lower perioperative risk, although recurrence of gallstone ileus has been reported in up to 5-8% of cases [2,4]. In this case, the surgical team performed a successful enterolithotomy without bowel resection or fistula repair, as no cholecystoenteric fistula was identified intraoperatively.

Anticipating this complication during ERCP was pivotal in ensuring timely diagnosis and intervention. In our patient, the endoscopist’s procedural note flagged concern due to the size and difficulty of stone extraction. This highlights the value of procedural foresight, documentation, and interdisciplinary coordination [7].

Ultimately, a multidisciplinary approach involving gastroenterology, surgery, and radiology is key to managing such complex scenarios. Post-ERCP monitoring should include not only signs of typical complications but also delayed presentations such as bowel obstruction in high-risk patients.

## Conclusions

This case highlights gallstone ileus as a rare but important complication following ERCP, even in the absence of a cholecystoenteric fistula. Large stones may fail to pass through the gastrointestinal tract, resulting in mechanical obstruction. Recognition of this risk, even without evidence of fistula, is crucial. Careful pre‑ and post‑procedural evaluation, procedural foresight, and clear documentation can aid in early diagnosis and prompt surgical management, ultimately improving patient outcomes. This case underscores the importance of vigilance in patients with large CBD stones undergoing ERCP.
